# Prevalence and factors associated with Posttraumatic Stress Disorder seven years after the conflict in three districts in northern Uganda (The Wayo-Nero Study)

**DOI:** 10.1186/s12888-015-0551-5

**Published:** 2015-07-24

**Authors:** James Mugisha, Herbert Muyinda, Peter Wandiembe, Eugene Kinyanda

**Affiliations:** 1 Makerere University, Child Health and Development Center, School of Health Sciences, Makerere Hill, Kampala, Uganda; 2 Butabika National Psychiatric Referral Hospital, Off Old Port Bell, 2958, Kampala, Uganda; 3 Sør-Trøndelag University College, E. C. Dahls gate 2, 7012 Trondheim, Norway; 4 Department of Statistical Methods, Makerere University, COBAMS Makerere Hill, Kampala, Uganda; 5 MRC/UVRI Uganda Research Unit on AIDS, Uganda/MRC-DFID African Leadership Award, 50-59 Nakiwogo Street, Entebbe, Uganda; 6 Department of Psychiatry, Makerere University College of Health Sciences, School of Health Sciences Makerere hill, Kampala, Uganda

**Keywords:** Posttraumatic stress disorder, Post conflict northern Uganda, Associated factors

## Abstract

**Background:**

Research on the prevalence of Posttraumatic Stress Disorder (PTSD) is still limited in low income countries yet PTSD can be a public health problem in post conflict areas. In order to respond to the burden of PSTD in northern Uganda, an area that experienced civil strife for over two decades, we need accurate data on its (PTSD) prevalence and the associated risk factors to facilitate public mental health planning.

**Methods:**

This study employed a cross-sectional study design and data collection was undertaken in three districts in northern Uganda: Gulu, Amuru and Nwoya. Respondents were aged 18 years and above and were randomly selected at community level. A total of 2400 respondents were interviewed using a structured questionnaire in the three study districts. In this study, multivariate logistic regression was employed to analyze the associations of socio-demographic factors, trauma related variables and the outcome of PTSD.

**Results:**

The prevalence of Posttraumatic Stress Disorder (PTSD) in the study population was 11.8 % (95 % CI: 10.5 %, 13.1 %) with a prevalence of 10.9 % (95 % CI: 9.3 %, 12.5 %) among female respondents and 13.4 % (95 % CI: 11.2 %, 15.7 %) among male respondents. Quite a number of factors were strongly associated with PTSD. Overall, a respondent had experienced 9 negative life events. In a multivariate logistic regression, the factors that were strongly associated with PTSD were: exposure to war trauma events, childhood trauma, negative life events, negative copying style and food insecurity. The findings also indicate no association between sex, age and PTSD.

**Conclusion:**

The prevalence rate of PTSD in the study communities is unacceptably high. Quite a number of factors were associated with PTSD. Effective public mental health services are needed that combine treatment (medical) psychological and social welfare programs especially at community level to address the high burden of PTSD. Longitudinal studies are also recommended to continuously assess the trends in PTSD in the study communities and remedial action taken.

## Background

Studies on prevalence of Posttraumatic Stress Disorder (PTSD) in low income countries are quite few as compared to the developed world [3, 7, 12, 40]. However, the studies that have been conducted in both high income and low income countries indicate that war and political violence are associated with high rates of PTSD [7, 12, 24, 38]. The prevalence rates in areas that have suffered from political conflict have ranged from zero percent (0.0 %) in conflict affected areas in Iran to 99 % in Sierra Leon [37]. Studies conducted in Uganda during active conflict reported rates of PTSD that varied between 18 % and 54 % [13, 27–29, 33, 39]. Some cross-sectional studies undertaken in Africa indicate that PTSD can still be a public health concern several years after the civil conflict [30, 32, 34] while Kuwert et al. [19] in a study conducted in German makes a similar observation. There are however few studies [33, 39] on the prevalence of PTSD and its associated factors in northern Uganda (especially those focusing on the general population) yet, this information is important for the planning of services to address post-conflict mental health conditions (including PTSD).

Northern Uganda went through one of the worst civil conflicts in the world that lasted over two decades [1, 6, 25, 28, 29, 33, 41]. The civil conflict was between the Uganda government forces and the Lord’s Resistance Army (LRA) rebels. Over 2 million people were displaced into internally displaced people’s camps (IDPs) due to the violent threats that involved rape, torture, mutilation, destruction of property and many of them [people] including children were abducted. For example, Vinck et al. [39] found an abduction rate of 44 % in a population based survey while Annan et al. [2] in their study among the males youth in the same region found a third of them reporting histories of abduction. The abductees were forced to commit atrocities against each other and their own communities including family members. They were forced to kill and/or witnessed shooting and were subjected to a lot of other inhuman acts [1]. These war experiences can significantly contribute to mental illness [1, 2, 4] including PTSD. In addition to the war experiences mentioned above, due to the direct and indirect consequences of war, many families in northern Uganda remain impoverished even after the war, with inadequate food, poor health care, limited chances for education among other vulnerabilities [7, 13, 15].

Now it is seven years since the cessation of hostilities between the government forces and the LRA rebels and efforts are being made to rebuild mental health services. These efforts are however, being hampered by the lack of up to date information on the state of the mental health of the population including information on the burden of war related PTSD at community level. This study attempts to address this deficiency by providing mental health data (including data on PTSD) seven years after the conflict.

Previous studies on war related PTSD have documented the following as some of the common risk factors: number of negative life events [1, 12, 33], exposure to a traumatic event [12, 13, 33], absence of basic social goods and services such as food [1, 13, 33], gender [30, 32–34], having a mental illness [15] and type of traumatic event [1, 33] among other factors. However, most of the studies on risk factors of PTSD were carried out on populations in active conflict, and therefore there is need to investigate the risk factors of PTSD in the post-conflict situation.

This study will undertake to address some of the methodological limitations of previous studies where PTSD screening tools but not diagnostic categories were used to define ‘PTSD caseness’ and where small community samples were used [28, 33]. Overall, the study aim was to assess PTSD prevalence and factors that are associated with higher PTSD prevalence. Further it aimed at assessing the comorbidity of PTSD with other common mental health conditions in three districts in post conflict northern Uganda.

## Methods

### Study design and setting

This study was conducted in three districts in northern Uganda: Gulu, Nwoya and Amuru. This community survey was part of a baseline study for a project delivering a novel community-based intervention to increase access to mental health services in post-conflict northern Uganda. The intervention uses surrogate kinship relationships; the Wayos (aunties) and Neros (uncles) to deliver community based mental health services. The project is supported by a grant from Grand Challenges Canada and Bill & Melinda Gates foundation. Within each district, with the help of local leaders, sub-counties were rated on how badly (intensely) they were affected by the 20-year civil conflict; from the most affected to the least affected. Two sub-counties, rated as most affected and that have health center(s) of at least level three (within the Ugandan health system), from each district were selected for the community-based intervention project. A baseline survey was conducted with a representative sample of adults from all the selected sub-counties. The sub-counties selected for this study were Lalogi and Koro in Gulu district; Amuru and Atiak in Amuru district; and Koch Goma and Alero in Nwoya district.

### Sample size and sampling plan

The sample size for the project baseline survey was computed to ensure a precision of at least 2.6 % in estimates of the proportions of community members with different mental disorders with a statistical significance level of 5 %. The disorders considered included major depression disorder (MDD), post-traumatic stress disorder (PTSD), alcohol dependency, moderate-to-high suicidality and generalized anxiety. Due to the clustered sampling method used, a design effect of 2.0 was assumed. Using the formula given in Kirkwood and Sterne [16] the largest sample size was 2400 adults that were required to attain a 2.6 % precision in the estimation of the prevalence of MDD. Previous studies had shown prevalence of MDD to be 29.3 %. The precise estimation of the prevalence of PTSD required a sample size of only 1933 adults. We used the sample size of depression since it is the common disorder reported in northern Uganda [33, 39] and this study being a baseline survey for an action research study, we needed to target more than one condition in addition to PTSD. Our project, in addition to PTSD, targets depression, suicide and anxiety disorder.

Respondents were selected through a two-stage cluster sample, stratified on the sub-counties. Study respondents had to be residents in the six sub-counties and aged 18 years or above. In each sub-county a parish was randomly selected and, a random sample of 400 individuals was taken of individuals from all the villages within it. Since there was no reliable source of information on the number of households in each village, a sampling frame based on households was not possible. Using the Health Centre III or IV as the central point in each parish, teams of research assistants fanned out in the 4 directions of the compass to interview study respondents. All adult members (18 years and above) in homesteads lying along a village road in a given direction of the compass were visited and interviewed. Interviews in each parish were conducted until a total number of 400 study respondents had been attained. In 2 sub-counties, the selected parish could not provide all the required 400 respondents so data collection was extended into the neighboring parish of the same sub-county. Because we were working with community leaders, we did not experience respondents refusing to take part in the interview. Each interview lasted between 50–70 min. Study interviewers were all psychiatric nurses who were fluent in the local dialect (Luo) and registered with the Uganda Nursing Council. They were assessed by the research team for competence in mental health before their training. They were trained by the research team for one week in objectives of the study, consenting procedures, administration of the study questionnaire and the referral process for those respondents found to have significant psychiatric problems. They were re-assessed for competence in relation to the study needs at the end of the training by the research team. In addition to the above, short debriefing sessions were conducted on a daily basis in the evening at the end of fieldwork to share experiences and challenges faced during the day. At the end of each debriefing session, data cleaning (checking on errors and completeness of data) could be conducted.

### Data collection tools

A structured questionnaire consisting of various standardized assessment tools previously employed in the Ugandan context by one of the investigators (EK) [15] was used in this study. The different assessment tools were translated into the local Luo language by a team of mental health professionals and also independently back-translated into English. A consensus meeting was then held to resolve the items where there was a wide disparity in the two back-translations from the originals.

*The key sections of the study questionnaire were:*

1. Socio-demographic factors: sex, age, marital status, highest educational attainment, religion, and employment status.
2. Psychosocial factors: (i) ‘Food insecurity’ (assessed by the question, ’in the last month, did you or your family have enough food?’); (ii) ‘Negative life events index’, constructed from items of the adverse life events module of the European Para-suicide Interview Schedule [14] that has previously been modified for the Ugandan situation by Kinyanda and colleagues [15]. The sub-scales of this index were derived as the number of events experienced (through summating at five items) and included war physical trauma events, sexual trauma events and war psychological trauma event sub-scales. Their corresponding α-Cronbach measures of reliability were 0.74, 0.66 and 0.65, respectively. iii) Adverse life events in childhood was assessed using the Childhood Trauma Questionnaire-Short Form (CTQ-SF) [5]. This is a 28-item questionnaire that asks about negative life events experienced as a child and as an adolescent. The respondent is required to respond based on a 5 point Likert format where 1 = ‘never true’; 2 = ‘rarely true’; 3 = ‘sometimes true’; 4 = ‘often true’; 5 = ‘very often true’. A total score was generated. To ensure that all the items of the CTQ-SF are scored in the same direction, the following items were reverse scored 2, 5, 7, 10, 13, 16, 19, 22, 26, and 28. The α-Cronbach of this scale in this study was 0.89; iv) Positive Coping style index. This was assessed using the Mental Adjustment to Cancer Scale (MAC) [5] whose items have been previously adapted to the Uganda situation [15]. Each of this scale's 17 questionnaire items is scored on a 4-item Likert scale 1 = (definitely does not apply to me), 2 = (does not apply to me), 3 = (applies to me),4 = (definitely applies to me). To score all the questionnaire items so that they are all in the same direction i.e., higher scores reflecting less negative coping style, questionnaire items that were cast negatively were reverse scored at analysis. A total score was generated so that higher scores reflected a more negative coping style, the α-Cronbach of this scale in this study was 0.93; v) Social support index was assessed using the Multidimensional Scale of Perceived Social Support (MSPSS) [8]. The MSPSS is a 12-item instrument that was designed to assess perceptions about support from family, friends and a significant other. Originally designed to have each item on this scale graded according to a 7 point Likert Scale, this was shortened to 4 choices namely, 1 = ‘strongly disagree’; 2 = ‘mildly disagree’; 3 = ‘mildly agree’; 4 = ‘strongly agree’; 7 = ‘if you very strongly agree’ as some choices did not make sense in the local cultural context. The α-Cronbach of this scale in this study was 0.92. vi) Self report on HIV status . This was assessed by asking the question, ‘what is your HIV status’.
3. Psychiatric disorders were assessed using the M.I.N.I. neuropsychiatric interview (MINI Plus) which is a modular DSM IV based structured interview [35]. The psychiatric disorder modules used in this study were: major depressive disorder, suicidality, alcohol dependency/ abuse disorders, generalized anxiety disorder and post-traumatic stress disorder. Family history of psychiatric disorder was assessed by the question, ‘has any member of your family (immediate family- parent, sibling, child) ever been diagnosed with a psychiatric disorder?’

### Statistical analysis

Data was analyzed using STATA software (StataCorp LP, 4905 Lakeway Drive, College Station, TX, USA). Logistic regression models were used to assess univariate associations between the dependent variable (PTSD) and the independent variables including exposure to war trauma variables, childhood trauma, and other negative life events including food insecurity and HIV positive status, age, sex and family history of psychiatric disorders. In total 12 independent variables (IVs) were considered at univariate analysis. Survey analyses using the ‘svyset’ command and the ‘svy’ prefix in STATA were used to account for a multi-stage cluster sampling design of the study including stratification, unequal selection probabilities, and clustering. All factors associated with PTSD at a level of significance of ≤0.1 in the univariate models were entered into a multivariable model to determine their independent associations with PTSD. The IVs were dropped from the multivariable logistic model if all their associated p-values were greater than 0.1 and there was no large reduction in Akaike Information Criterion (AIC). Sex and age were taken as apriori confounders and were retained in the final model.

### Ethical considerations

Approvals were obtained from Makerere University College of Health Sciences Science and Ethics Committee. We also secured ethical approval from the Uganda National Council for Science and Technology. Study approvals from all participating district authorities were also secured. Informed consent was obtained from all study respondents after explaining the objectives and procedures of the study. Informed consent was secured from the respondents before they could be subjected to the study tools. Written consent was secured from those (respondents) who could read and write. They could fill out a consent form which had been translated into the local dialect. For respondents who could not read and write, we used verbal consent. In addition to the verbal consent, they could put their thumb print on the consent paper. Study respondents found to have significant psychiatric problems were referred to the health centre III or IV in their sub-county. The parent interventional study had helped establish a psychiatric service in all health centre III and IV’s in the survey sub-counties. Respondents were assured of confidentiality before the start of each interview. All data collected were kept under key and lock and only accessible to the research team. Personal identifiers were not used at data entry.

## Results

### Study population

Data collection was carried out between 2^nd^ January 2013 and 2^nd^ June 2013. A total of 2406 respondents were interviewed (819 from Amuru district, 770 from Gulu district and 817 from Nwoya district). During analysis, a total of 45 records were excluded because of missing data on key variables. The excluded records did not differ significantly from those retained on district, age and sex.

### Characteristics of study respondents

Sample characteristics are provided in Table 1. Of the 2361 respondents sampled, 1475 (62.5 %) were females; 555 (24 %) were aged between 18 and 24 years; 672 (29 %) were aged above 45 years; and 1617 (69 %) were married/cohabitating. Additionally, the majority of the respondents (1399, 59.4 %) had only attained primary level education. Just like in other regions of the country, majority of the respondents were peasant farmers at 2141 (90.8 %).

**Table 1:** Sample socio-demographic characteristics (The Wayo-Nero Study in Northern Uganda)

Potential factor	Number in study (N, %)
Sex	
Male	1475 (62.5)
Female	886 (37.5)
Age group	
18 – 24	555 (23.5)
25 – 34	644 (27.3)
35 – 44	490 (20.8)
45 – 54	672 (28.5)
Marital status	
Currently married/cohabiting	1617 (68.9)
Widowed	289 (12.3)
Separated/Divorced	172 (7.3)
Single	269 (11.5)
Education level	)
No education	559 (23.8)
Primary	1399 (59.7)
Secondary and above	386 (16.5)
Religious affiliation	
Catholics	1846 (78.5)
Protestants	296 (12.6)
Others	211 (9)
Employment	
Other	220 (9.3)
Peasant farming	2141 (90.7)

### Exposure to war traumatic events and other negative life events

Results on exposure of the respondents to the war traumatic events and other negative life events are presented in Table 2. Participants were exposed to a mean of 4.0 war-related stressing events. At least 52 % experienced physical traumatizing events; 79 % psychological traumatizing events and 8 % experienced sexual events. Higher proportions of men as compared to women experienced physical traumatizing events (58.0 % vs 46.0 %; p < 0.001) and psychological traumatizing events (81.5 % vs 76.1 %; p < 0.001). However, more women than men experienced sexual traumatizing events (9.7 % vs 4.0 %; p = 0.004). Overall, a respondent had experienced on average 9 negative life events.

**Table 2:** Exposure to war related traumatic events and non-war related negative life events among the study respondents (The Wayo-Nero Study)

Potential factor	Number in study (N, %)	Female (N, %)	Male (N, %)
War physical trauma no. of events			
No event	1161 (48.4)	799 (54.1)	362 (42.1)
1 - 2 Events	788 (32.8)	555 (36.0)	233 (24.3)
3 - 5 Events	451 (18.8)	163 (9.9)	288 (33.6)
War psychological trauma no. of events			
No event	493 (20.5)	339 (24.0)	154 (18.2)
1 - 2 Events	777 (32.4)	556 (36.4)	221 (24.1)
3 - 6 Events	1131 (47.1)	622 (39.7)	509 (57.7)
War sexual trauma no. of events			
No event	2218 (92.4)	1368 (90.5)	850 (96.1)
1 -2	110 (4.6)	88 (5.8)	22 (2.5)
3 - 4	73 (3.0)	61 (3.8)	12 (1.4)
Cumulative no. of war traumatic events			
None	427 (19.2)	288 (20.4)	139 (16.8)
At least one event	1934 (80.8)	1741 (31.7)	193 (21.6)
At least three events	1121 (47.2)	569 (47.9)	552 (61.6)
No. of non-war related negative life events			
1-5	777 (32.3)	454 (30.8)	323 (35.8)
6-10	710 (29.5)	454 (29.9)	256 (28.9)
11+	919 (38.2)	612 (39.3)	307 (35.4)
Childhood trauma index			
Low (<33^th^ percentile)	703 (29.2)	461 (28.6)	242 (26.1)
Medium (33-67^th^ percentile)	874 (36.3)	546 (36.5)	328 (37.6)
High (>67^th^ percentile)	829 (34.5)	513 (34.9)	316 (36.3)
Food security in the past month			
Enough	979 (43.1)	571 (39.7)	408 (47.3)
Not enough	1,290 (56.9)	852 (60.3)	438 (52.7)
Negative event Coping Style index			
Low (0–3)	479 (19.9)	364 (23.3)	115 (12.7)
Medium (4–10)	705 (29.3)	453 (29.8)	252 (29.4)
High (11–20)	1222 (50.8)	703 (46.9)	519 (57.9)

Furthermore, 815 (35.5 %) experienced high traumatic events during their childhood and 554 (23.0 %) were from families with psychiatric disorder history.

### Prevalence of Post-Traumatic Stress Disorder (PTSD) and co-morbidities

The prevalence of Post-traumatic Stress Disorder (PTSD) in this population was 11.8 % (95 % CI: 10.5 %, 13.1 %) with a prevalence of 10.9 % (95 % CI: 9.3 %, 12.5 %) among female respondents and 13.4 % (95 % CI: 11.2 %, 15.7 %) among male respondents (Table 3). Of the individuals with PTSD, about 39 % had generalized anxiety disorder, 53 % had MDD, 21 % had moderate to high tendency for suicidality, and about 12 % had alcohol dependency.

**Table 3:** Comorbid Psychiatric disorders for PTSD in northern Uganda communities (The Wayo-Nero Study)

	Overall prevalence		Prevalence among PTSD patients	
Condition	N	%	n	%
GAD	349	14.6	113	38.6
Alcohol abuse	160	6.9	31	11.8
Alcohol dependency	157	6.6	33	12.2
MHS	178	7.6	61	21.4
MDD	582	24.8	149	52.7

### Potential factors associated with Post-Traumatic Stress Disorder (PTSD)

At univariate analysis, exposure to war-related traumatic events, childhood trauma, experience of other negative life events, food insecurity, and HIV status were significantly associated with PTSD. In particular, exposure to high number of war-related traumatizing events, whether physical, sexual or psychological events, was associated with at least seven times the odds of having PTSD. Sex and age were also associated with PTSD (Table 4). Education level, employment status, religious affiliation and marital status were not associated with PTSD at univariate analysis and therefore were excluded from further analyses.

**Table 4:** Potential factors associated with PTSD in northern Uganda (The Wayo-Nero Study)

Factor	PTSD (n, %)	OR (95 % CI)	*p*-value	Adjusted OR^a^ (95% CI)	*p*-value
War physical trauma no. of events					
No event	55 (4.8)	1.00		1.00	
1 - 2 Events	88 (11.4)	2.63 (1.89, 3.66)	<0.001	1.34 (0.95, 1.88)	0.096
3+ Events	136 (30.8)	8.68 (6, 12.56)	<0.001	3.42 (2.16, 5.42)	<0.001
War sexual trauma no. of events					
No event	224 (10.3)	1.00		1.00	
1 - 2 Events	27 (24.8)	2.6 (1.63, 4.15)	<0.001	1.23 (0.73, 2.07)	0.439
3+ Events	28 (38.9)	5.65 (3.33, 9.61)	<0.001	2.02 (1.08, 3.76)	0.027
War psychological trauma no. of events					
No event	18 (3.7)	1.00		1.00	
1 - 2 Events	39 (5.1)	1.58 (0.76, 3.3)	0.221	1.37 (0.65, 2.89)	0.401
3+ Events	222 (20.1)	6.91 (3.44, 13.87)	<0.001	2.94 (1.53, 5.63)	0.001
Number of negative life events					
1-5	44 (5.8)	1.00		1.00	
6-10	75 (10.7)	1.96 (1.2, 3.2)	0.008	1.31 (0.87, 1.97)	0.194
11+	161 (17.8)	3.53 (2.45, 5.09)	<0.001	1.46 (1.05, 2.03)	0.026
Childhood trauma					
Low	45 (6.5)	1.00		1.00	
Medium	74 (8.6)	1.27 (0.86, 1.89)	0.233	1.21 (0.83, 1.77)	0.326
High	161 (19.8)	3.4 (2.29, 5.06)	<0.001	2.28 (1.49, 3.48)	<0.001
Negative Coping Style index					
Low	116 (24.8)	1.00		1.00	
Medium	79 (11.4)	0.4 (0.27, 0.59)	<0.001	0.50 (0.3, 0.81)	0.006
High	85 (7.1)	0.24 (0.18, 0.33)	<0.001	0.33 (0.22, 0.49)	<0.001
Social support					
No	181 (15.6)	1.00			
Yes	99 (8.3)	0.52 (0.39, 0.69)	<0.001		
Family history					
No	197 (11.1)	1.00			
Yes	79 (14.3)	1.34 (0.91, 1.98)	0.134		
HIV status					
Negative	243 (11.7)	1.00			
Positive	34 (21.6)	2.09 (1.48, 2.95)	<0.001		
Sex					
Female	161 (10.9)	1.00		1.00	
Male	119 (13.4)	1.32 (1.11, 1.58)	0.002	1.09 (0.85, 1.39)	0.506
Age group					
18 – 24	50 (9)	1.00		1.00	
25 – 34	84 (13)	1.48 (1.06, 2.06)	0.020	0.96 (0.64, 1.43)	0.825
35 – 44	63 (12.9)	1.51 (1.09, 2.1)	0.014	0.81 (0.52, 1.26)	0.345
45 – 54	83 (12.4)	1.46 (1.1, 1.95)	0.009	0.78 (0.52, 1.16)	0.217
Food security					
Yes	72 (7.4)	1.00		1.00	
No	204 (15.8)	2.38 (1.85, 3.05)	<0.001	1.74 (1.33, 2.28)	<0.001

^a^For each variable we adjusted for all other variables in the column

At multivariable analysis, the odds of having PTSD did not differ significantly between sexes and age groups (Table 4). Individuals who were exposed to war traumatic events and related stress were more likely to have PTSD (Table 4). The higher the events that were experienced the higher the odds of having or sustaining PTSD. After adjusting for socio-demographic characteristics, childhood trauma and experience of other life negative events individuals who experienced at least three war-related physical traumatic events had thrice the odds of having PTSD (adjusted OR (aOR) = 3.42; 95 % CI: 2.16, 5.42). Similarly, individuals who experienced at least three psychological traumatizing events had thrice the odds of having PTSD (aOR = 2.94; 95 % CI: 1.53, 5.63).

High childhood trauma was also independently associated with PTSD. Individuals with high childhood trauma were more than twice likely to have PTSD compared to those who did not have trauma (aOR = 2.28; 95 % CI: 1.49, 3.48). Further, individuals with medium or high copying styles against negative life events or trauma were more likely to have recovered or have no PTSD.

Food insecurity was also independently associated with PTSD (aOR = 1.74; 95 % CI: 1.33, 2.28).

Gender and age were not associated with PTSD. It is likely that the impact of trauma events did not differ much across gender and age.

## Discussion

War is complex and can have long lasting trauma on the population [19]. This is the first population based study to focus on the prevalence of PTSD in post conflict northern Uganda, seven (7) years after the civil conflict. The major finding in this study is that the burden of PTSD in the study communities is high. And, public mental health programs that are well coordinated should respond to the high prevalence of PTSD in the study communities. Reducing the burden of mental disorders including (PTSD) is vital since mental disorders can interfere with the normal functioning and productivity of community members [41].

Our findings however, give a prevalence rate that is lower than that reported in previous studies undertaken in northern Uganda (e,g Roberts et al. [33] reported a prevalence rate of 54 % while Vinck et al. [39] reported a prevalence rate of 44.5 % of PTSD among their study population). The possible factors to explain the high prevalence rates of PTSD in the earlier studies could be: a) because they were undertaken during active conflict and b) the studies referred to above used self- report screening instruments to determine the prevalence of PTSD in the study population. Kuwert, et al. [19] argues that determining prevalence of PTSD using self- report screening instruments should be considered presumptive. However, using screening instruments during active conflict could be understandable given the fact that it may be difficult to mobilize enough mental health workers to undertake diagnostic assessment c) spontaneous remission of PTSD especially if the number of traumatic events experienced was low [18, 20, 22] d) the impact of public health intervention in the study communities, for example, the activities of civil society organizations.

This study was conducted using a DSM IV criteria performed by means of face to face interviews undertaken by trained clinicians and therefore more likely to give us more realistic prevalence rates in the region. In some respondents, PSTD symptoms could have persisted sevenyears after the conflict possibly because there were no well coordinated and effective medical, psychological and social welfare programs in the study communities [7, 12, 28]. War is likely to affect the functional capacity of government and other service agencies to deliver public mental health services [7, 17].

Elsewhere in Africa, in a study that used diagnostic assessment to determine the prevalence of PTSD in a post conflict area, a prevalence rate of 37.4 % was reported in Algeria and 15.8 % in Ethiopia [12]. Though the findings in Algeria quite differ much from our findings, the findings in this study are comparable with those of Ethiopia. Studies conducted elsewhere in the West in post conflict areas indicate a range between 15 % and 24 % of PTSD [12]. Some studies that have been conducted among refuge population in the past indicate almost the same prevalence. For example, Mollica et al. [21] estimated a prevalence rate of 15 % of PTSD among refugees in Thai-Cambodia boarder [21] while ElSarraj et al. [9] found a prevalence rate of 20 % of PTSD among survivors of torture in Gaza.

Our findings indicate an association between PTSD, MDD, anxiety and attempted suicide. Pfeiffer & Elbert et al. [29] noted that this comorbidity is always the norm rather than exceptional. Other studies conducted in post conflict areas in Africa indicate an association between PTSD symptom severity and other mental health conditions such as MDD, anxiety and suicidal behavior [32, 34]. It would be important that public health planners put in place adequate and skilled manpower to address these comorbidities and put in place adequate resources (such as drugs) for their effective management [23]. But this is challenge to countries such a Uganda where mental health is less of a priority as compared to other sectors such as malaria and HIV/AIDS.

Despite the low prevalence rate reported in this study as compared to the prevalence rates reported in earlier studies conducted in active conflict [33, 39], there are some commonalities between the studies conducted during active conflict and our current study. Just like in Roberts et al. [33] and Vick et al. [39] it is still clear in this study that extreme exposure to traumatic events is associated to the outcome of PTSD. Our respondents experienced physical, psychological and sexual trauma events. Our findings indicate that respondents suffered more psychological events (79 %) as compared to physical (58 %) and sexual events (8 %). The type of physical, psychological and sexual trauma events reported in this study don’t differ much from those reported in other studies conducted in the same region [33, 39, 41]. Interventions in northern Uganda need to deal with these trauma events as one of the ways of reducing the burden of PTSD among post conflict communities.

We report in this study that childhood trauma was associated with PTSD. Pfeiffer et al. [29] noted that in northern Uganda, complex trauma exposure could happen repeatedly among the formerly abducted children and young adults for a long time and this had implications on cumulative exposure and their mental health (the abducted children and young adults). In some of our respondents, the onset of PTSD could have been during their childhood/adolescence [29]. However, this does not mean that during their life trajectories, some individuals may be predisposed to PTSD, including other psychiatric conditions. This could be due to genetic or environmental exposure.

In this study, negative life events were associated with PTSD. Similar finding are reported in other studies [28, 29, 39] conducted in northern Uganda. The events reported in this study are those that had happened six months before the survey and include lost of a parents(s), having a sick parent (s), lost of a sibling(s), having a sick/ill sibling among others. Other studies conducted elsewhere [18, 30, 32, 34] indicated that the risk to trauma increases with number of negative events experienced. The precarious nature of positive war conditions is likely to make the impact of these events stronger among the study communities.

Not having enough food (food insecurity) was associated with PTSD. Hadley et al. [11] in their study in rural Ethiopia noted that food insecurity contributes to stressful life and both food insecurity and stressful life events were independently associated with symptoms of high posttraumatic stress in their study. Similar findings are reported by Schaal et al. [34] in their study in Rwanda among orphans and widows. As communities continue to settle in northern Uganda, they seem to be struggling with basic survival, including stress associated with finding adequate food.

In terms of coping, negative coping style was associated PTSD. Though the link seems not to be direct, it has been argued that negative coping style consequences may exacerbate posttraumatic stress symptoms by promoting negative coping strategies [36] such as alcohol abuse and high risk sex behaviour. Similar findings are reported by Read et al. [31] and Greening et al. [10]. Interventions designed to impact on negative coping may help to some extent offset this risk to PTSD in the study communities [11] and are recommended.

While other studies [29, 33] have reported on age and sex being strongly associated with PTSD, we did not find such associations in this study. The possible reason to explain why there were no age and gender differences associated with PTSD in the study areas is that the impact of trauma events could have been almost similar across age and gender.

## Conclusion

We studied the prevalence and risks factors of PTSD in a population based study in post conflict northern Uganda. Our major finding is that the prevalence rate of PTSD is high in the study area. These findings call for government interventions that can effectively address the, medical, social and psychological wellbeing of the people in the study communities. PTSD was associated with GAD, alcohol dependency, alcohol abuse and MDD. These findings have paramount clinical importance to health workers working in the region. The clinician has to pay attention to quite a range of conditions and, it is their role as clinicians to explore whether the patients have all the symptoms if effective care is to be provided [19]. If some conditions remain unaddressed, it will be hard to effectively reduce the prevalence of PTSD in the region. Public health planners therefore should appreciate that PTSD symptoms could result from different determinants [12] and therefore should be able to avail adequate resources for comprehensive care for all the related (comorbid) conditions. Overall, these findings provide useful baseline information for our Wayo-Nero project in terms of prevalence, comobirdity and risk factors of PTSD in the study communities. This information is useful for planning and programming of interventions on mental health in the study communities including PTSD.

### Study limitations

This study adopted a cross sectional design and therefore it is difficult to determine the direction of causality of the factors associated with PTSD. Being a correlation study, we are not able to report on risk factors. Our focus remains on the factors associated with PTSD in the study communities. PTSD is often a chronic disorder following a traumatic event and therefore longitudinal studies are likely to produce more conclusive information on the prevalence and associated risk factors for PTSD than cross-sectional studies such as this one. We also did not collect information on the percentage of our respondents that had benefited from psychosocial interventions in the study area. This information could have improved our understanding why the prevalence rate of PTSD reported in this study is lower than that reported in previous studies. This limitation should be addressed in future studies.

Given the low education level of our respondents, it became hard for some respondents to remember the number of events seven years after the conflict. The events reported in this study could have been lower than the actual events experienced. We also depended on verbal reports from respondents to determine past history of psychiatric disorder in their family yet family members may not have accessed mental health services for a diagnostic interview/assessment. However, given the fact that over 80 % Okello [26] of Ugandans do not access mental health services, diagnosis assessment of past psychiatric history would be difficult to get and one has largely to dependent on verbal reports.

We used Psychiatrists nurses to administer the study tools on the respondents. Though this is quite unavoidable given the fact that there are few mental health workers in the region of higher qualifications (by the time this study was conducted, northern Uganda had only three psychiatrists and out of the three, two had retired and were only working with the University in Gulu). The region also had one clinical psychologist). It is plausible that more accurate data on diagnosis assessment would have been obtained if we had used highly trained cadres in mental health. We however, used a support team of psychologists and psychiatrists from Makerere University to supervise data collection and offer regular training .
